# Ethical issues associated with HIV molecular epidemiology: a qualitative exploratory study using inductive analytic approaches

**DOI:** 10.1186/s12910-019-0403-9

**Published:** 2019-10-07

**Authors:** Farirai Mutenherwa, Douglas R. Wassenaar, Tulio de Oliveira

**Affiliations:** 1 School of Applied Human Sciences, University of KwaZulu-Natal, Private Bag X01, Scottsville, Pietermaritzburg, 3209 South Africa; 2 KwaZulu-Natal Research Innovation and Sequencing Platform (KRISP), School of Laboratory Medicine and Medical Sciences, University of KwaZulu-Natal, Durban, South Africa; 3 Department of Global Health, University of Washington, Seattle, USA; 4 Centre for the AIDS Programme of Research in South Africa (CAPRISA), Durban, South Africa

**Keywords:** Molecular epidemiology, HIV phylogenetics, HIV network research, Data sharing, In-depth interviews, Informed consent, People with HIV/AIDS, Privacy/confidentiality, Qualitative methods

## Abstract

**Background:**

HIV molecular epidemiology is increasingly recognized as a vital source of information for understanding HIV transmission dynamics. Despite extensive use of these data-intensive techniques in both research and public health settings, the ethical issues associated with this science have received minimal attention. As the discipline evolves, there is reasonable concern that existing ethical and legal frameworks and standards might lag behind the rapid methodological developments in this field. This is a follow-up on our earlier work that applied a predetermined analytical framework to examine the perspectives of a sample of scientists from the fields of epidemiology, public health, virology and bioethics on key ethical issues associated with HIV molecular epidemiology in HIV network research.

**Methods:**

Fourteen in-depth interviews were conducted with scientists from the fields of molecular epidemiology, public health, virology and bioethics. Inductive analytical approaches were applied to identify key themes that emerged from the data.

**Results:**

Our interviewees acknowledged the potential positive impact of molecular epidemiology in the fight against HIV. However, they were concerned that HIV phylogenetics research messages may be incorrectly interpreted if not presented at the appropriate level. There was consensus that HIV phylogenetics research presents a potential risk to privacy, but the probability and magnitude of this risk was less obvious. Although participants acknowledged the social value that could be realized from the analysis of HIV genetic sequences, there was a perceived fear that the boundaries for use of HIV sequence data were not clearly defined.

**Conclusions:**

Our findings highlight distinct ethical issues arising from HIV molecular epidemiology. As the discipline evolves and HIV sequence data become increasingly available, it is critical to ensure that ethical standards keep pace with biomedical advancements. We argue that the ethical issues raised in this study, whether real or perceived, require further conceptual and empirical examination.

## Background

Phylogenetic analysis is a system of computational methods used to study how organisms are genetically related to each other. The process involves inspecting genetic material extracted from different sources and identifying evolutionary relationships. The evolutionary relationships are presented in the form of a phylogenetic tree [1], which looks like a family tree and represent historical and hypothetical relationships.

Phylogenetic analytic techniques have been widely used to study the history of HIV, including how it got into humans (where and how) and its spread across nations and populations. This is done by examining the relatedness of different viruses and how they evolved from a common ancestor [2]. Two viruses are regarded as more related if they share a more recent common ancestor and vice-versa. HIV phylogenetics has great potential to advance our knowledge of HIV epidemics by providing high quality data on the diversity of HIV strains and on HIV transmission dynamics at country and global levels.

The use of HIV phylogenetic analysis for molecular HIV surveillance and for optimizing HIV prevention strategies through targeting specific populations at high risk of transmitting HIV is gaining prominence [3–5]. Customarily, HIV sequence data are obtained from HIV positive patients as part of routine clinical care, for example, to prescribe an antiretroviral treatment regimen during diagnosis and when drug resistance is suspected [6]. Some sequences are obtained from study cohorts, primarily for research purposes. After phylogenetic analysis of HIV sequences, networks in which HIV transmission is occurring are identified, which inform the design of high impact HIV prevention and care interventions for persons in those networks. Targeted interventions may include follow-up of potential transmitters and partner notification [5], provision of HIV testing services and pre-exposure prophylaxis as well as linkage and re-engagement to care [7]. Although common in high-income countries (HICs), which can afford the high costs and have the requisite laboratory infrastructure for genotyping, molecular HIV surveillance and HIV phylogenetic studies are inevitable in low-and middle-income countries (LMICs) as the cost of genotyping becomes affordable [8].

In spite of the conventional use of HIV genetic sequence data for research and public health surveillance, guidance on use is unclear [9–11]. Specific guidance for use is particularly important considering that analysis of HIV phylogenetic sequences is data intensive. Sequences may be collected at one site, but stored for long periods, analysed or re-linked with data sets from elsewhere. Researchers have therefore expressed concern over potential ethical and legal issues that could arise from the application of HIV phylogenetics in HIV transmission dynamics research and in public health settings [9, 10, 12, 13]. However, despite the numerous ethical concerns raised, conceptual and empirical studies to explore these issues are relatively scarce [14, 15].

This paper reports on an exploratory study of scientists’ views on key ethical issues associated with HIV molecular epidemiology as a contribution to the small but growing body of empirical studies on this topic. An earlier analysis of the same dataset using a predetermined analytical framework found that favourable risk-benefit ratio and informed consent were the most invoked ethical principles [16]. To the best of our knowledge, this is the first study to explore the views of experts on this important emerging field of significant public health importance, using inductive analytical methods. As HIV sequencing becomes increasingly affordable, and the need to optimize HIV prevention methods becomes a necessity [17, 18] in generalized epidemics, the ethical issues associated with these techniques will assume greater importance and require conceptual and empirical research.

## Methods

This paper reports on a sub-part of a broader project which explores ethical issues associated with HIV molecular epidemiology. The broader study consists of three sequential work packages, which began with a desk review reported elsewhere [10] intended to guide the development of a conceptual framework. This was followed by the current study - an empirical investigation of perceptions of experts on key ethical issues associated with HIV molecular epidemiology. The final work package [in preparation] will explore the perspectives of community representatives’ understanding of HIV phylogenetic research.

### Sampling

Twenty-nine experts were purposively selected using non-probability sampling strategies. First, we used the following keywords to identify authors published in peer-reviewed journals: HIV phylogenetics, molecular epidemiology and ethical issues. Potential interviewees were also identified through the current authors’ research and professional networks. Invitations to participate in the study were emailed between September and October 2016. Each invitation was accompanied by a consent script, which was read just before each interview to obtain verbal informed consent.

Of the 29 scientists, 15 confirmed availability for the interview. Ten did not respond despite follow-up emails. Although the remaining four responded to the invitation, they indicated that they were not comfortable to be interviewed on the topic and proposed other scientists whom they regarded as more informed about HIV phylogenetics. Participants were from eight countries in three continents: Africa, Europe, and North America.

### Data collection

Fourteen interviews were conducted between November and December 2016 guided by an open-ended interview schedule. Each interview was different from the others as subsequent questions were guided by the responses from each preceding question. Participants were first asked a broad question on their knowledge and experience with HIV phylogenetics followed by specific questions on what they perceived to be key ethical issues associated with the technique. The intention was to maximize diversity of views without confining participants to any pre-defined themes. All interviews were conducted by the first author (FM), a social scientist with a postgraduate degree in research ethics, but no formal training in molecular epidemiology. Each interview lasted an average of 45 min.

Due to the wide geographic distribution of the participants, most interviews were conducted via Skype. All participants provided verbal consent for both the interview and digital recording of the interview. Verbal consent was requested instead of written consent because of practical challenges associated with obtaining written consent from participants remotely (participants were from different parts of the world). This was explained in the protocol, which was approved by the Biomedical Research Ethics Committee of the University of KwaZulu-Natal, South Africa (Ref BE224/16). The participants were also not a vulnerable population and the study questions were entirely scholarly and required no disclosure of personally sensitive information or any other information that could identify the participants.

### Data analysis

All interviews were transcribed verbatim by Shuvai Machingura (SM), a Social Scientist, who was not involved in the data collection. Transcripts were checked by FM for accuracy. We applied the constant comparison approach [19], which is based on grounded theory [20]. The approach allowed us to identify and generate themes that helped address the research questions in a systematic way while leaving an audit trail. The approach has four main stages, as follows.

### Open coding

FM together with SM read the first four transcripts in detail to become familiar with the contents and summarized what each respondent said using a word or phrase that best captured the meaning of each bit of the text.

### Progressive focusing

As a team (FM and SM), we looked at the collection of codes and discussed and grouped them together into meaningful categories taking note of any subcategories that emerged. The categories were developed based on what the respondents reported as the most important ethical issues as well as ideas that helped us to structure or explain their views and experiences. The collection of categories formed our initial coding frame. The initial coding frame and data analysis commenced during the initial stages of data collection to ensure that ideas that emerged during data analysis could be explored in subsequent interviews.

### Applying the coding frame

The coding frame was programmed into NVivo and all the transcripts were imported into the software. The coding frame was applied systematically across each transcript on a code-by-code basis. New themes and novel ideas of understanding different perspectives were explored in subsequent interviews. The coding frame was also revised to accommodate any emerging issues, taking note of such changes and the reason for the change. Coding was done by both SM and FM and reviewed by DW at the time of writing. Discrepancies were resolved by mutual agreement. The coding frame was continuously updated to accommodate new information until no new insights relevant to the research question were provided by additional interviews.

### Summarizing and interpreting findings

The last stage involved exploring relationships and patterns in the issues raised by respondents and summarizing insights which contributed most effectively to achieving our research objectives.

## Results

One of the interviews could not take place due to internet connectivity challenges. The findings presented in this section are therefore based on 14 interviews from the following participants: ethicists (5), epidemiologists (3) virologists (3) a geneticist (1), a medical anthropologist (1) and a public health practitioner (1). The profiles of the participants are summarized in Table 1.

**Table 1:** Participant profiles

Participant number	Skills	Country	Gender
1	Clinical and Epidemiological Virology, HIV Phylogenetics	Belgium	Female
2	Public Health Ethics, Epidemiology, Research Ethics	Sweden	Male
3	Genetics, Genomics, Next Generation Sequencing, Health Research Ethics	South Africa	Female
4	Global Justice and Bioethics, Global Health and International Research Ethics	USA	Male
5	Infectious Disease Epidemiology, HIV, Molecular Epidemiology	Uganda	Female
6	Bioethics, Research Ethics, HIV Prevention, Clinical Trials	USA	Female
7	Applied Ethics	USA	Male
8	Medical Anthropology, HIV Prevention, Research Ethics	Malawi	Female
9	Molecular Epidemiology, Genetics, Microbiology, Infectious Diseases Epidemiology, Research Ethics	USA	Male
10	Molecular Virology, Molecular Epidemiology, Bioinformatics	Uganda	Male
11	Evolutionary Biology and Bioinformatics	United Kingdom	Male
12	Genetics, Sequencing, RNA, DNA	USA	Male
13	Research Ethics, Bioethics, Governance	New Zealand	Female
14	Infectious Diseases, Biology and Epidemiology of HIV Transmission, Molecular Epidemiology	USA	Female

Four broad themes, capturing the key ethical concerns associated with molecular epidemiology were inductively derived from the data: (i) Consent, (ii) risk and benefits, (iii) privacy and confidentiality and (iv) public health versus research continuum. We include selected quotations to highlight themes raised. In the following section, we discuss each of these themes in turn, followed by an overall discussion of the ethical issues raised.

### Informed consent

Participants were concerned about difficulties in explaining molecular epidemiology to research participants and other stakeholders. It was noted that those outside the discipline might also find it hard to appreciate key scientific concepts, especially the inferences that could be made from the data and the level of certainty of the scientific findings in identifying those associated with risk of transmitting HIV. One of the experts in HIV phylogenetics remarked,

*I think it’s an impossible task to properly explain. ...I wouldn’t know how to simply explain it to prospective participants. Even in court it takes a long time to explain all this. In a clinical set-up there is not that time, you know, or in public health circumstances there is not enough time to explain anything and even if there is enough time, the patients will never grasp what exactly it means, what can be done and what cannot be done. (#1).*

*...if you want to have informed consent one of the key areas is the understanding of the topic. And I think by now genetics is becoming so complex that you even don’t understand it. ... so the question in this is how are you going to explain to participants etc. The consent has to remain informed. So information could be the next key challenge to either researchers or to the institutions that are employed as researchers to make sure that communication becomes much better. (#3)*

*So there is a challenge to not just be a good scientist and ask people if they are willing to participate. There is an extra barrier to explain what you can and what you can’t learn by sequencing HIV? (#7)*

One of the misunderstandings anticipated by our participants was whether HIV network research determines directionality of transmission. They cautioned against the delivery of messages that could potentially mislead the community.

*“It is not helpful if a researcher says in public that, with phylogenetic analysis they know who infected whom, while they mean that as a statistical phrase meaning that, on average, if you do have an analysis, you can actually make a profile of a person who is infecting someone else”. (#1)*

*... it is difficult to say that by virtue of the science we are doing, we are potentially identifying individuals who potentially transmitted to one another. We are exposing them to potential criminal statutes should that information become available. And again it is particularly difficult when all we are doing is saying that they are putative or potential transmission partners. We cannot in anyway prove that they are transmission pairs. (#14)*

Participants noted that misrepresentation of HIV molecular epidemiology could have a negative effect on study recruitment as it could potentially deter prospective participants from engaging in related research. In view of the negative social consequences associated with positive HIV status, people living with HIV were seen to be more likely to be sensitive to, and critical of, the information they receive about HIV network research studies as the results could have negative implications for their lives. For example, if HIV patients were in the audience*,* they might say*,*

*“aah now they have techniques that can tell who infected who, and then if this kind of disease goes around in the community, the patients won’t wanna take part in research anymore”. (#1)*

The absence of clear cut-offs for HIV transmission clusters was also identified as an area of concern considering that clusters form the foundation of HIV network research. One of the experts in HIV phylogenetics said:

*Yeah, that is a very difficult issue. … because in the end everything is a transmission cluster from the first patient that was infected with HIV up to everybody who is infected now. ... so where do you put the border? That is still a discussion.... So the definition of a transmission cluster depends on what you wanna do with the data and that is not entirely clear for some people.(#1)*

### Risks and benefits

Participants expressed strong views that both scientists and laypersons may not fully know or understand the potential risks associated with HIV molecular epidemiology or be able to accurately quantify the probability and magnitude of a specific risk occurring, using currently available information. Similar concerns were raised about benefits.

*But I don’t think they are going to understand the degree to which the data generated has the potential, some day as technology progresses, ... to take away some of the autonomy. At the same time the data are powerful that maybe is the thing that informs us to be able to make the vaccine, which everyone would like to see happen successfully. And we can’t know all the risks of anything that we do. And so I think there is a difficult tension between benefits and risk. (#12)*

*Yes it’s a complicated thing... So actually, we did a lot of studies here. We try to explain phylogenetics to some of the population here and what we will try to do with the data. And we found that it was indeed very difficult for people to understand the process, the benefits and the risks associated with it.* (#9)

*I think we are all working in good faith and we developed consent so that the situation will be working. But I think the idea of truly getting consent from someone and the moment they get consent they truly understand the risks, I think that’s a fallacy. I think what we are doing is say that we trust the people doing this work to work in the best interest of the population and protect individuals to the extent that we can. But there are risks that we do not anticipate and we have to make sure that the people who use the data downstream understand the protection that should be applied to this data. (#12)*

*So if you are working in this area you probably got to explain some things that are a bit complex. But I hope it will make sense that there is a tremendous irony here that the people, who might have introduced these research studies, don’t understand the risks. It’s too complicated. (#7)*

It is one thing to provide information and yet another for that information to be understood. One of the key recommendations provided by participants for ensuring that participants understand the information they are given was to test for comprehension.

*“Basically you have to test their understanding. I think that is the only way we have at least a little chance of being sure that the issues are better understood. (#7)*

### Privacy and confidentiality

Most interviewees acknowledged that HIV molecular epidemiology was a powerful tool which could provide finer details about HIV transmission dynamics than traditional epidemiological techniques. Participants were, however, concerned about privacy violations, privacy harm and threats to confidentiality associated with the analysis of HIV sequence data. Specifically, participants were concerned about risks to identifiable groups despite the acknowledgement that HIV phylogenetic data are ordinarily anonymized. The concerns were more pronounced within the context of data sharing and publication of HIV sequence data in the public domain.

*But the issue then becomes how we shared this data with anyone including how we published that data, and that, I think becomes the real issue. The science is only meaningful if we are able to share with others within the field and that’s where it becomes very difficult. How did you share the data without compromising the privacy of the people whose data contributed to the science? (#14)*

*...who gets access to this data and how long will that be accessible and in terms of data sharing policies who is going to be responsible in future...#8*

*I think what we are also realizing is that most of the work that is being done on HIV phylogenetics, especially in research settings or even for public health, they actually get that information from people who are coming in for genotyping where they are looking at drug resistance. So they probably have the clinical and demographic information of that particular person and then they can now make some inferences and I think there is that potential challenge of even privacy violations and perhaps stigma...(#3)*

*“I think the biggest issue is that when we study small or large populations of genetic sequences of HIV, we know a lot about the people who contribute to those sequences and the relationship between them. We learn a lot about the people who contribute to the sequences depending on the amount of associated attributes that we have for the sequences... but I think it is potentially possible even without identifying information to work backwards with the other information to identify accurately who the infected individual is” (#14)*

The power of HIV molecular techniques to identify individuals and social groups associated with high HIV transmission events was considered a key driver of HIV-related stigma. Participants argued that existing stigma associated with HIV in some settings could be further aggravated through insensitive reporting of the scientific findings.

*“... you can link your analysis quite strongly to the community where you collected the sample and that can have normative implications or negative normative implications. The people can feel stigmatized. For instance in the case of HIV we have got this example where there are comments about HIV originated in this part of Africa and those were considered or perceived to be stigmatizing by people who live in those areas. (#3)*

Although strong views were expressed that, currently, HIV phylogenetics cannot by itself prove exact individual transmission, there was a perceived fear among participants that information, communication and technology tools might facilitate the identification of research participants in HIV network studies. Such information could potentially be abused.

*Yeah, as long as the information that you ask belongs to a big enough group then it’s impossible to actually, as a researcher, link a particular sequence to a particular individual. Where it becomes difficult is when patients themselves release too much information on social media, for example. And if someone really wants to identify someone it might be possible.... I can imagine that if there is too much social information that along with a lot of information that the patient is releasing outside the research question, for people who really want, they might be able to identify a person, but still you cannot prove transmission, that is who infected whom. You cannot prove that.” (#1)*

*And I think to me the most critical information is the fact of the potential to infer putative transmission, which is to say, if two individuals have linked genetic sequences, meaning they are nearly genetically identical, identical enough that we could infer what we call putative transmission it is possible that one individual transmitted to another. That could put those individuals in a difficult position if that information were made available to almost anyone and I very clearly try to use the term putative, because this data do not come out to prove transmission. (#14)*

*In Uganda for example sex work is illegal, it is criminal... homosexuality is also illegal. Then of course, as you know in many countries Uganda inclusive, intentional transmission of HIV is also a crime, ... definitely this causes legal concerns. #10*

A recurring concern shared by most interviewees was limited understanding among scientists and researchers of the risks to individual privacy associated with HIV molecular epidemiology techniques.

*“... people have many reasons why they would be worried about privacy and confidentiality but they don’t have an easy way to understand what is that risk with this technology. #7*

*I think it remains a potential risk only as long as people don’t realize what the risks are. And once people start to realize that these data hold within them a lot more information than they realized, only then will people start to realize what they can do with those data and how they can be both used and thus misused. So my hope is that no one will ever use this data to go searching for opportunities to criminally prosecute an individual for HIV transmission. Having said that, do I think it could happen? Sure. It could. But right now I don’t think it’s likely to happen mostly because I don’t think people have really thought through that. I don’t think that’s high on people, certainly in the research field, that is not high on anybody’s list, but again I think once, at least in the field of research, it becomes a potential risk. (#14)*

*We could explain these risks to them and they will say “this is a risk, I don’t want to participate”, but in reality, in the real world where we would have explained this it’s a risk of perceptions instead of a risk of reality. The perception is that this information can directly implicate somebody in having infected someone else but the reality is that it’s not true. But people’s perceptions, can be seen as most important than realities. (#7)*

Some participants recommended the use of *differential privacy (*a strategy commonly used to reduce the risk of privacy breaches related to health care data) to mitigate privacy violations in HIV molecular epidemiology. However, those with more experience with both differential privacy and HIV phylogenetic research expressed reservations about the proposal, citing procedural challenges in its application as summarized by the following quote:

*We have been trying to look at the strategy ... and probably the simplest thing I can say is that it is a very challenging strategy applied to molecular epidemiology. Because the structure of the network is critical to the interpretation of the results and differential privacy relies on the introduction of noise. The purpose for introduction of certain amount of noise into the data set is so that you preserve the structure of the data set but you make it less possible for the user of the data set to identify key features of identifiers of individuals in the data set and I don’t mean names. I just mean identifiers of any kind. And the challenge that I have with it is the introduction of noise almost by definition corrupts the structure of the data set. So I think we are not done with it but I will say it’s not something that in my mind is easily adapted to molecular epidemiology. (#14)*

### Public health versus research

Participants acknowledged that boundaries for the use of HIV sequence data are currently not clearly defined. In their view, a thin line exists between use of HIV sequence data for surveillance, research and for clinical care. Concerns were raised that if not properly managed, this could result in serious abuse of samples and related criminal and civil rights issues.

*Actually I had a court case in* ..., *which was exactly on the wrong side of this line. So this was a public health effort where they had an agreement with the hospital to track the transmission process. The hospital uncovered the names of these people to the public health workers. In the end, this became a court case and all these people in this cluster were suing one other person of the cluster because the public health workers didn’t know enough about what can and cannot be done. (#1)*

*...but I think it is the use of the data and it is not just criminal uses. It is people using the data for very laudable reasons pursuing the mission of the public health department, which is to improve the health of the local community, which could in fact backfire if not done in the right way. But I don’t know what the right way... (#14)*

Participants argued that public health goals were not always in line with those of medical care. Consequently, the trade-off between privacy and efforts to mitigate the burden of the epidemic would need to be carefully balanced.

## Discussion

The aim of this study was to explore the perspectives of experts on key ethical issues associated with HIV phylogenetics. Our analysis revealed that while participants were generally optimistic about the use of HIV phylogenetics for research and public health, they were concerned that communicating the concepts and procedures involved in molecular epidemiology in ways that could easily be understood by prospective participants would be challenging. In addition, there were misgivings about the capacity of researchers and research participants alike to fully delineate the risks and benefits associated with this technique. More specifically, while there was consensus that HIV molecular epidemiology poses a risk to individual privacy, the nature and quantum of the risk could not easily be determined.

Our participants expressed concern that researchers would find it difficult to communicate effectively about HIV molecular epidemiology - in particular HIV network research. They also envisioned that research participants in HIV phylogenetic studies might find it difficult to appreciate the conceptual and methodological issues associated with these molecular techniques. Our findings are consistent with results from a USA study that assessed stakeholders’ perceptions of molecular epidemiology studies. The study showed that most participants lacked a clear understanding of the potential applications of HIV molecular data, which ultimately affected their perceptions of the risks and benefits associated with HIV network research [14]. Furthermore, the study also showed that participants’ views of the topic changed over the course of the interview as participants received new information.

Misunderstandings were described, despite a significant amount of time spent by researchers to explain HIV network research to participants using different approaches incorporating visual aids. Such misunderstandings may reflect lower literacy levels and respondents’ naivety about scientific concepts as observed in previous studies conducted in low to medium income countries [21, 22]. Questions need to be asked about the appropriate level at which to discuss phylogenetic research with prospective research participants and how to ensure that the research messages do not unduly deter them from participating in phylogenetic research due to limited understanding or frank misunderstanding.

Challenges are expected when communicating technical and scientific terms like genetics or genomics partly due to the non-availability of such words in the vernacular language in which informed consent is obtained [21–23]. However, researchers are obliged to take additional steps to explore, develop and implement appropriate strategies for conveying research messages, to maximize understanding, notwithstanding the difficulties outlined above. Among low literacy populations, simple vernacular language and analogies from everyday local examples have been applied successfully to clarify scientific terms commonly used in clinical trials [24]. Several strategies have also been used in consent documents to explain genetics to prospective research participants by linking them to commonly used terms like heredity or inheritance and how observable individual characteristics or conditions are passed from parents to their offspring [25]. Similar approaches could be applied to explain the genomics of pathogens, with explanations adapted for the evolution of viruses and HIV network research. The provision of verbal and visual explanations could enhance both comprehension and retention of the disclosed information [26].

Drawing from the findings from our study and the available literature, future studies could use deliberative approaches during the consent process for HIV phylogenetic studies. This could allow prospective research participants to express their fears and concerns while experts correct any myths and misconceptions about the technique. Tests of understanding could possibly also be routinely implemented [27]. Educational programmes for the general public on the power and pitfalls of HIV phylogenetics might also be helpful to allay any spurious fears and concerns that prospective participants may harbour. The area of HIV phylogenetics research could also be an interesting case study in research ethics because of the distinct ethical issues it raises.

Molecular epidemiology involves data and sample sharing. The concept of data sharing is not only abstract but also unknown to most individuals and communities outside the sciences. Comprehensive guidelines on access, sharing and use of HIV sequence data should be developed (or revised if available) in consultation with key stakeholders cognizant of the potential risks of harm that may result [9, 28]. Recently, the World Health Organisation developed new ethics guidelines on the ethics of public health surveillance [29, 30], which could be reviewed to incorporate HIV genetic data for public health surveillance. However, for effective guidelines to be developed, the real and perceived risks of harm associated with the technique need to be clearly defined. This would require further research, broader consultation and community engagement. It is one thing to develop a set of guidelines and a governance framework and yet another to implement and enforce them. Without a clear strategy on enforcement and compliance, it would be difficult to control or minimise any privacy-invasive use of HIV molecular data.

A key component of the informed consent process is whether participants understand the risks and benefits of the study, failure of which undermines the likelihood of obtaining truly informed consent [14]. There is an increasing risk that people living with HIV could potentially withdraw from HIV testing and treatment programmes and research [28] out of fear of unintentional negative social and legal consequences of being associated with HIV transmission.

Our analysis showed some gaps in understanding of what constitutes privacy risk associated with HIV molecular epidemiology, its properties and boundaries. This is not surprising. Risk to individual privacy is commonly identified as a key ethical issue in most genomic studies [31] and in viral genetic studies on HIV transmission dynamics [13, 15]. With advances in technology the nature of privacy, and by extension, privacy harm, has evolved significantly from what it was known to be in the past decade. De-identification and data anonymisation, traditionally regarded as cornerstones of protection of individual privacy in research, have increasingly become illusory in genomic research. Advances in technology and data access from the internet have considerably simplified cross-matching of datasets and chances of re-identification [31].

Without a clear delimitation of privacy risk and privacy harm, it would be hard to identify privacy and security controls required to protect participants’ data and the limitations and risks that may remain despite the availability of such measures [32]. In addition, it would be difficult to rule out privacy harm when it occurs or to identify novel forms of this harm should they emerge. Risk-minimization strategies could also be difficult to formulate [15].

Some participants noted that HIV genetic data might result in serious civil and criminal cases depending on how the data are managed. This finding suggests that guidelines on the use of HIV sequence data should be responsive to privacy issues. Furthermore, the boundaries within which HIV phylogenetic data are used could be blurred [9, 27] as the field and its applications evolve. These findings call for further ethical deliberations on the downstream use of HIV sequence data for purposes other than the original intended use - HIV diagnosis and treatment. Ordinarily, public health surveillance data are collected without the express consent of the patient [33, 34]. Further careful deliberation on the requirements for waiving of consent or use of broad consent for secondary use of clinical data, HIV genetic sequences in particular, might be needed.

Molecular epidemiology is a relatively new scientific discipline characterised by active methodological development. The changing nature of privacy is also increasingly recognised as an ethical challenge, particularly the difficulty of anonymising genomic data. Discussions on privacy become pertinent as specific population sub-groups have been subjected to prosecution [35] while some ethnic communities have either been stigmatised or blamed for their perceived role in spreading HIV. The controversial Haitian HIV connection [36, 37] is a classic example.

Several strategies could be implemented to protect research participants and build public trust in HIV phylogenetic network research. One approach could be to systematically investigate whether existing ethics, legal and governance frameworks are responsive to the privacy concerns raised in this paper. Secondly, RECs reviewing molecular epidemiology studies could benefit from the expertise of data protection and privacy experts, particularly information and computer technologists and legal experts. Lastly, the use of differential privacy as a data security strategy to mitigate privacy breaches in HIV network research [15] needs validation to ensure optimal use in the field of molecular epidemiology.

HIV molecular surveillance makes use of HIV sequence data from HIV positive persons who have agreed to HIV testing and drug resistance genotyping primarily for treatment purposes. Such clinical data could be linked to surveillance and patient follow-up without the express consent of the sample donor [6], which brings into focus the long-standing tension between public health, ethics and human rights. Questions need to be asked about whether HIV positive persons understand what they are consenting to when they give their samples for HIV genotyping and the implications of their consent [9, 38]. The rationale would be to identify the most appropriate models of consent for HIV molecular epidemiology studies and to optimize the public health and research utility of HIV genotype data without violating research ethics guidelines and standards.

### Limitations

The findings of our study were based on in-depth interviews conducted with a sample of scientists with backgrounds in epidemiology, public health, virology and bioethics. Most of our participants were from Europe and USA. The views expressed by our participants may not, therefore, represent the full range of perspectives from experts in global HIV molecular epidemiology. Different issues may arise from experts working in other geographical, cultural or socio-economic regions that were not represented in our sample (Asia and Latin America), which could be an opportunity for future follow-on research. However, the primary aim of our study, like many other qualitative studies, was not to make generalizable inferences about the population of experts from the purposive sample of scientists. Rather, we sought to explore perspectives on the subject, which could be further explored or validated using quantitative approaches.

We are also aware that some researchers and scientists from high income countries may also run collaborative HIV phylogenetic projects in other parts of the world. A typical example of such partnerships is the Phylogenetics and Networks for Generalized HIV Epidemics in Africa (PANGEA-HIV) consortium, which is an international collaborative project which seeks to use viral sequence analyses to assess HIV in Africa [12]. The views of our respondents may not, therefore, apply only to their own home regions because of their exposure to other research settings. Furthermore, the amount of HIV phylogenetic research has not been uniform across countries and the geographical divide. Relatively fewer HIV phylogenetic studies have been conducted in Africa, with the majority having been conducted in high income countries [39].

Some participants had a strong background in research ethics but had limited exposure to HIV molecular epidemiology and vice-versa. A few had been exposed to both research ethics and molecular epidemiology. Our respondents might have over- or underestimated the benefits and risks of HIV molecular epidemiology depending on their area of specialization. Experts in molecular epidemiology might have expressed conservative views about the risks to privacy associated with HIV phylogenetics because of limited ethics knowledge. On the other hand, those with a strong research ethics background might have overestimated the predictive power and accuracy of HIV phylogenetics and consequently become overly concerned about unlikely scenarios, threats to privacy, stigma and related ethical and legal concerns.

Molecular epidemiology research involves multiple stakeholders with differing interests. Among them are researchers who generate the data, research participants from whom the sequence data and associated attributes are collected, health professionals involved in HIV care and treatment and the general public. This report focused primarily on one group of stakeholders — the scientists. The views of historically stigmatized populations, for example, men who have sex with men, sex workers, injecting drug users and people living with HIV would also be critical for a fuller appreciation of these discussions. Their views could be different from those expressed by experts interviewed in our study and actively soliciting their views could enrich and consolidate some of the ethical concerns raised. Members of Research Ethics Committees as well as Data Access Committees also have a critical contribution to make towards a fuller appreciation of the risks concerned.

## Conclusion

Based on the interpretation of the findings and our previous work, we conclude that existing research ethics guidelines may need to be adapted to the distinct ethical issues arising from HIV molecular epidemiology, in particular, phylogenetic HIV network research. As the discipline of molecular epidemiology evolves with the increasing availability of HIV sequence data it is critical to ensure that research ethics guidelines keep pace with biomedical advancements in the field. In particular, the social and public health implications of HIV molecular epidemiology studies need to be constantly interrogated in order to understand the impact and implications of HIV molecular epidemiology studies’ results on individuals and communities. Furthermore, the design of novel and evidence-based models of community engagement, consent and research governance could be instrumental in advancing both the science and ethics of the molecular epidemiology of HIV.

## Data Availability

The datasets generated and analysed for the study reported in this paper are not publicly available due to confidentiality requirements. However, they can be requested from the corresponding author in an anonymized format after consent is obtained from participants.

## Abbreviations

HICs: High-income countries
HIV: Human immunodeficiency virus
LMICs: Low and middle-income countries
RECs: Research ethics committees
